# The short term impact of uncomplicated cataract surgery on retinal layers thickness

**DOI:** 10.3389/fmed.2025.1537402

**Published:** 2025-07-21

**Authors:** Marco Gioia, Maddalena De Bernardo, Aniello La Marca, Martina De Luca, Sergio Pagliarulo, Mariagrazia Avella, Alfredo Mignone, Nicola Rosa

**Affiliations:** Department of Medicine, Surgery and Dentistry “Scuola Medica Salernitana”, University of Salerno, Baronissi, Italy

**Keywords:** cataract surgery, cataract, retina, retinal thickness, OCT, SD-OCT, retinal layers

## Abstract

**Objective or purpose:**

To detect the short-term impact of cataract surgery on retinal layers thickness, as the exact mechanism of fundus changes after phacoemulsification has not yet been fully clarified.

**Design:**

A retrospective observational study.

**Subjects, participants, and/or controls:**

Seventy eyes of 70 patients with age ranging from 49 to 92 years, scheduled for cataract surgery, were included.

**Methods, intervention, or testing:**

All subjects underwent a complete ophthalmological examination, including ART-OCT volume with Heidelberg Spectralis before and approximately one month later cataract surgery.

**Main outcome measures:**

The macula was divided into a central foveal region and four parafoveal regions (superior, inferior, nasal, temporal). The scans were then automatically segmented into the different retinal layers and the changes in each layer were assessed.

**Results:**

The results revealed that both the inner retinal layers and the entire retina exhibited a statistically significant thickening in foveal and parafoveal region: IRL (*p* < 0.001), ONL (*p* < 0.001), GCL (*p* = 0.010), RNFL (*p* = 0.020), and ALL (*p* < 0.001). Conversely, the outer retinal layers showed a statistically significant reduction in thickness only within the parafoveal regions: ORL (*p* < 0.001).

**Conclusion:**

This study may provide a pathophysiological explanation for post-phacoemulsification changes affecting the retina.

## Introduction

Cataract is the leading cause of treatable vision loss worldwide, affecting approximately 20 million individuals, (1) and cataract extraction is the most frequently performed surgical procedure (2). Any invasive eye surgery, including cataract surgery, initiates a biochemical cascade resulting in an immunological response. Advancements in surgical techniques and pharmacological treatments have effectively reduced this response in recent years, thereby lowering the risk of postoperative complications such as posterior capsule tear, vitreous presence in the anterior chamber, and the development of cystoid macular edema.

Phacoemulsification is the most widely utilized surgical technique, recognized as a well-standardized and safe procedure (3) generally yielding favorable visual outcomes. However, it is not entirely risk-free, as it can lead to side effects impacting various ocular structures, including the retina. Research indicates that cataract surgery can be a contributing factor in the development of posterior segment complications, including cystoid macular edema in pseudophakic patients, potential onset of age-related macular degeneration, and worsening of diabetic retinopathy (4–8). Even uncomplicated cataract surgery can induce retinal changes that are not detectable through ophthalmoscopic examination, often leading to unnoticed damage and subsequent decline in visual function (9–11).

The advent of optical coherence tomography (OCT) has enabled increasingly detailed imaging of retinal and choroidal structures (12, 13). This method is straightforward, safe, non-invasive, and highly precise, allowing for macular measurements with a resolution of 8–10 micrometers (14). Modern OCT equipment also permits precise and detailed segmentation of retinal layers. OCT has become a fundamental tool for identifying subclinical alterations affecting the macula after cataract surgery, leading to a growing number of studies in this area.

Previous research has largely focused on examining modifications across the entire retinal area, with somewhat conflicting results, (15–22) or on changes affecting specific retinal layers involved in common postoperative complications [e.g., outer plexiform/inner nuclear layers in Irvine Gass syndrome; (21–23) retinal nerve fiber layer (RNFL) in elevated IOP (24)].

In 2018, for the first time, Kurt and Kı*l*ıç (25) published a study that focused on the segmentation of all retinal layers and individually analyzed the alterations following phacoemulsification. Their findings revealed non-uniform thickening across different retinal layers (25). The present study aims to analyze retinal segmentation thickness before and one month after cataract surgery to detect any short-term surgically induced changes in each layer.

## Materials and methods

This research study was conducted in accordance with the ethical guidelines outlined in the Declaration of Helsinki, and the necessary approval from the Institutional Review Board (IRB) (CECS, South Campania Ethics Committee, protocol no. 16544) was secured. All individuals gave a written consent to take part in the study.

Subjects with lens opacities, eligible to cataract surgery at the Ophthalmology Unit of the University of Salerno, which underwent uneventful cataract surgery with in the bag IOL implantation, were recruited for this retrospective observational study.

Patients with corneal leukoma, epiretinal membrane, diabetic retinopathy, advanced hypertensive retinopathy (grade II to IV), age-related macular degeneration, central serous chorioretinopathy, glaucoma, macular hole, previous laser treatment or intravitreal injections, presence of vitreomacular disease, previous ocular surgery, optic neuropathy, uveitis, uncontrolled hypertension with medication, autoimmune diseases, glycated hemoglobin A1c (HbA1c) level > 6.5% and ocular or systemic conditions that could cause retinal alterations were excluded. Only patients with nuclear or cortical opacities that allowed the preoperative OCT examination were recruited.

Therefore, 70 eyes of a total of 70 subjects (42 female and 28 male patients), aged 49 to 92 years (mean age 73 ± 9 years), were selected.

### Clinical and instrumental assessment

During the preoperative visit, the participants undertook a complete ophthalmological examination, which involved assessing their clinical history, measuring their uncorrected and corrected visual acuity using the Snellen chart, conducting slit-lamp biomicroscopy and anterior segment evaluation, determining their IOP with a Goldmann applanation tonometer, performing fundus examination, and measuring their axial length using an IOLMaster 500 device (Carl Zeiss Meditec AG, Jena, Germany, version 5.4.4.0006), and spectral domain OCT scanning (Spectralis; Heidelberg Engineering; Heidelberg, Germany, version 6.0) using the “automatic real-time” (ART) volume program. One month later (an interval between 28 and 31 days), the OCT evaluation was reperformed using the device’s follow-up mode.

### Surgical technique

Before surgery, a Mydriasert^®^ tablet (Théa Pharma) (containing a combination of tropicamide and phenylephrine hydrochloride, 0.28 mg/5.4 mg) was located in the lower conjunctival fornix to promote mydriasis. Peribulbar anesthesia with 0.75% ropivacaine, followed by eyelids, eyelashes, and conjunctiva disinfection with 5% povidone iodine, and phacoemulsification with Constellation^®^ Vision System (Alcon Laboratories, Inc.) were performed.

All surgeries followed the established protocol, including emulsification of cataractous lenticular nucleus passing through a clear corneal main tunnel of 3 mm on the vertical axis and the “Divide and Conquer” surgical technique, by different surgeons.

In all cases, the same preloaded hydrophobic intraocular lens Tecnis^®^ Monofocal (PCB00) was implanted in the capsular bag. Injection of 0.1 mL/1 mg of cefuroxime (Ximaract^®^; Bausch & Lomb UK Ltd.) into the anterior chamber and closure of the main tunnel and two paracentesis were final steps of the surgery. In the postoperative period, the use of dexamethasone and levofloxacin 1 mg/ml + 5 mg/ml (Ducressa; Santen Italia S.r.l.) five times daily for one week, then diclofenac 1 mg/ml (Visunac; Visufarma S.p.A.) three times daily for the following three weeks were prescribed as post-surgical antibacterial and anti-inflammatory prophylaxis.

### OCT analysis: retinal parameters

**FIGURE 1:**
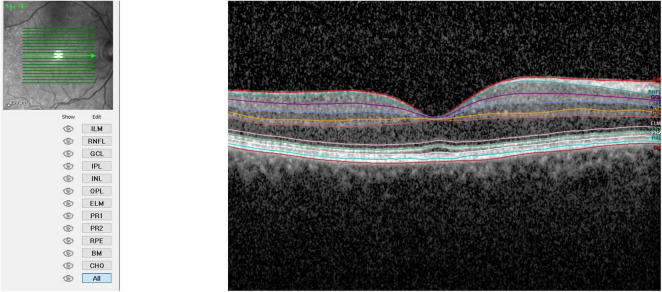
Retinal layers before cataract surgery.

**FIGURE 2:**
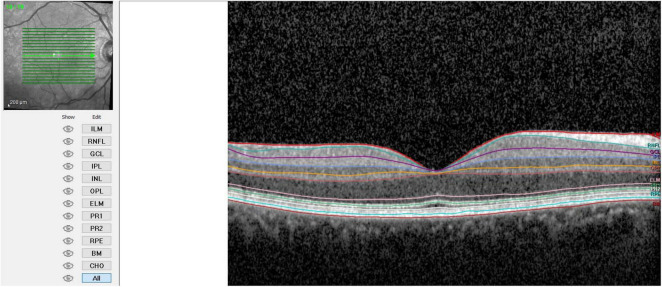
Retinal layer after cataract surgery.

**FIGURE 3:**
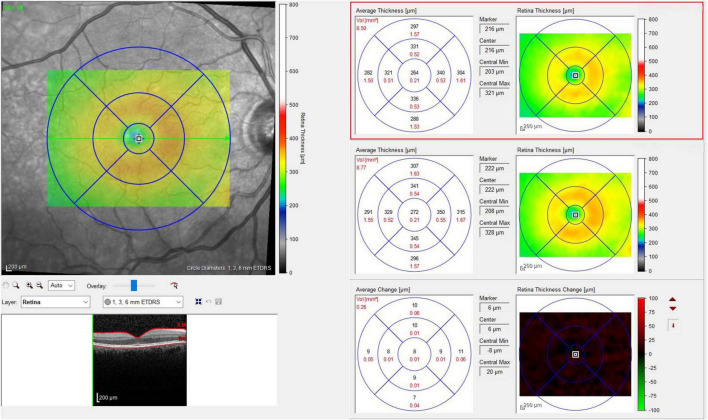
Thickness map before and after cataract surgery and differential thickness map.

The study involved the use of Spectral-Domain Optical Coherence Tomography (SD-OCT) with the Heidelberg Spectralis (Heidelberg Engineering, Heidelberg, Germany) to image participants’ retinas. The key details of the imaging process and analysis used are the following:

- Imaging Parameters: Scan Area: 20° × 15° (5.9 × 4.4 mm)
Frames: average of 100 frames using Automatic Real-Time (ART) mode
Sections: 19 horizontal scans spaced at 240 μm intervals
Resolution: 512 A-scans per B-scan
- Image Quality: images with a signal-to-noise ratio (SNR) less than 15 dB were excluded to ensure high-quality data.
- Retinal Layer Segmentation: the segmentation of retinal layers was performed automatically by the Heidelberg Spectralis software. The system identified 11 optical interfaces to study the 10 distinct retinal layers.
- Thickness Measurement: the standard Early Treatment Diabetic Retinopathy Study (ETDRS) grid was used. Measurements were taken at the following regions: fovea-centered Circle, 1 mm in diameter, and Parafoveal Regions, in superior, nasal, inferior, and temporal regions each with a 3 mm diameter. Finally, the average thickness of the retinal layers was calculated for the fovea (1 mm), parafoveal regions (3 mm each), and the combined average of these five regions (Figures 1–3).

This detailed protocol ensures consistent and high-quality retinal imaging and analysis, crucial for accurate assessment and study of retinal health and diseases.

Therefore, values were collected for total thickness (ALL), outer retinal layers (ORL), inner retinal layers (IRL), retinal pigment epithelium (RPE), outer nuclear layer (ONL), outer plexiform layer (OPL), inner nuclear layer (INL), inner plexiform layer (IPL), ganglion cell layer (GCL), and nerve fiber layer (NFL). IRL included the sum of the layers between NFL and external limiting membrane (ELM), while ORL included layers from RPE to ELM.

Photoreceptor layer (PRL) measurement resulted from the subtraction of RPE measurements from the ORL.

Then, the same trained ophthalmologist (M.D.B.) checked the automatic retinal layers segmentation, misalignments, decentration or motion artifacts and reviewed data extrapolated from collected OCT scans to avoid measurement biases.

The ETDRS grid was manually aligned with the foveal pit, if not correctly positioned.

All examinations were performed between 12:00 and 2:00 PM. All measurements were made using the Heidelberg Spectralis software (Heidelberg Engineering; Heidelberg, Germany, version 6.0).

### Statistical analysis

SPSS software (IBM SPSS Statistics version 25) was used to conduct statistical assessment.

The normality of data distribution was computed with the Kolmogorov–Smirnov test. All parameters of patients and controls between the preoperative and the control visits were compared using a two-tailed *t*-test, for data distributing in accordance with the Gaussian curve, and the Wilcoxon signed-rank test, for data distributing according to a different trend. Statistical significance was considered for a *p*-value of less than 0.05. To perform the analysis G*Power software (version 3.1.9.4) was utilized. To maximize the statistical power utilizing a paired *t*-test, the sample size with α error = 0.05, 1-β error = 0.95 and effect size = 0.437 was set. Non-centrally parameter δ = 3.656, critical *t* = 1.995, Df = 69, total sample size = 70 and actual power = 0.95 were then calculated. In addition, to maximize the statistical power utilizing Wilcoxon test, the sample size with α error = 0.05, 1-β error = 0.95 and effect size = 0.450 was decided. Non-centrally parameter δ = 3.68, critical *t* = 1.997, Df = 65.84, total sample size = 70 and actual power = 0.95 were calculated.

## Results

Table 1 provides a summary of the retinal layers’ measurements, whereas Table 2, illustrates the differences between pre- and post-surgical measurements, which can be summarized as follows:

**TABLE 1:** Thickness of retinal layers (μm).

		PRE						POST					
		Mt	3S	3I	3N	3T	3MT	mt	3S	3I	3N	3T	3MT
**ORL**	Mean	85	81	80	82	81	81	85	79	79	80	79	79
	SD	5.9	3.2	3.6	3.6	3.3	3.2	4.8	4	3.3	3.6	3.7	3.5
	Min	75	74	74	74	74	74	74	74	72	73	69	74
	Max	109	89	98	97	90	92	99	91	87	91	90	90
	Median	84	80	80	81	80	80	85	79	78	80	79	79
**IRL**	Mean	176	245	243	245	234	242	187	258	257	260	247	255
	SD	21.1	19	20	19.2	22	18.9	3.4	25	21.2	18	16.9	18.6
	Min	111	146	149	154	132	145	134	146	160	210	208	200
	Max	216	291	284	286	323	281	241	302	313	304	282	299
	Median	177	246	245	245	233	244	183	260	256	261	247	256
**EPR**	Mean	15	15	14	14	14	14	16	15	14	15	14	14
	SD	3.1	1.4	1.3	1.3	1.1	1	2.8	1.9	1.7	1.8	1.6	1.6
	Min	12	12	11	12	12	12	13	12	11	12	12	12
	Max	30	18	17	18	16	17	24	20	18	19	18	18
	Median	15	14	14	14	14	14	15	14	14	15	14	14
**ONL**	Mean	81	68	69	69	74	70	91	74	73	79	80	77
	SD	15.4	18.4	17.7	17.2	12.6	13	15.7	22.9	19.6	15.6	13	15.6
	Min	42	33	37	31	46	45	51	31	34	34	48	48
	Max	112	122	131	101	109	102	139	238	193	134	140	170
	Median	84	69	69	70	73	69	91	73	74	79	80	77
**OPL**	Mean	27	435	33	36	29	33	27	34	34	33	30	33
	SD	7.8	8.4	7.4	10.7	5.5	5	8.5	7.6	6.1	7.2	5.4	3.7
	Min	12	20	21	23	19	22	2	8	24	24	20	25
	Max	52	62	56	64	48	46	61	59	54	60	56	44
	Median	26	33	30	33	27	33	25	32	32	31	29	32
**INL**	Mean	25	41	42	43	38	41	24	42	42	42	39	41
	SD	8.8	4.51	5.9	5.7	3.8	3.4	7.3	3.9	5	4.1	4	3.1
	Min	12	20	21	23	19	22	2	8	24	24	20	25
	Max	56	55	71	67	47	49	46	50	65	54	48	48
	Median	23	41	41	43	38	41	23	42	42	42	38	38
**IPL**	Mean	19	36	35	37	36	36	20	3	38	39	38	38
	SD	3.9	4.8	5.9	5.1	5.3	4.4	3.9	4.8	4.8	4.9	5.1	4.3
	Min	13	17	19	21	23	23	11	17	17	24	23	25
	Max	33	51	47	48	52	52	31	51	51	49	51	50
	Median	18	39	37	38	36	36	20	39	39	39	39	39
**GCL**	Mean	14	45	44	43	39	43	14	49	48	47	43	47
	SD	4	8.2	9.6	7.2	7.6	7.2	4	8.1	8.2	7.8	7.8	7.2
	Min	7	21	14	24	16	21	7	9	20	25	17	23
	Max	25	68	68	60	60	64	25	70	70	67	68	68
	Median	14	46	46	44	40	44	14	50	49	46	42	47
**RNFL**	Mean	12	21	22	19	18	20	12	24	24	21	18	22
	SD	2.8	3.4	3.9	2.1	2.5	1.9	2.8	4	3.9	2.7	1.8	2.4
	Min	6	12	9	15	14	14	6	9	15	15	13	17
	Max	22	31	30	24	27	24	22	35	37	27	22	28
	Median	12	21	22	19	17	20	12	24	24	21	18	21
**ALL**	Mean	258	327	325	328	315	324	270	339	336	340	326	336
	SD	26.3	15.9	16.4	15.7	15.1	14.8	25.8	16.7	17.5	17.8	16.7	16.3
	Min	136	260	267	271	278	277	170	284	284	288	291	292
	Max	302	375	363	371	351	363	324	380	392	386	362	378
	Median	261	327	325	329	314	324	268	337	337	341	326	334

mt: thickness of retinal layers at the foveal location (1 mm diameter). 3MT: mean thickness of retinal layers at the macular location (3 mm diameter). 0.3S: retinal layers thickness 3 mm superior to the fovea. 3I: thickness of retinal layers 3 mm inferior to the fovea. 3N: thickness of retinal layers 3 mm nasal to the fovea. 3T: thickness of retinal layers 3 mm temporal to the fovea. ORL, outer retinal layers. IRL, inner retinal layers; RPE, retinal pigment epithelium; ONL, outer nuclear layer; OPL, outer plexiform layer; INL, inner nuclear layer; IPL, inner plexiform layer; GCL, ganglion cell layer; RNFL, retinal nerve fiber layer; ALL, total retinal thickness.

**TABLE 2:** Difference in thickness (μm) of retinal layers pre/post-operation.

		Δ PRE/POST					
		Mt	3S	3I	3N	3T	3MT
**ORL**	Mean	0	1	1	1	2	1
	SD	0	−1	0	0	0	0
	Min	1	0	2	1	5	1
	Max	10	−2	11	6	0	2
	Median	−1	1	2	1	1	1
	*P*	0.742	0.002	< 0.001	0.002	< 0.001	< 0.001
**IRL**	Mean	−11	−13	−13	−15	−13	−14
	SD	−2	−6	1	1	5	0
	Min	−23	0	−11	−56	−76	−55
	Max	−22	−11	−29	−18	41	−19
	Median	−7	−14	−11	−15	−14	−12
	*P*	< 0.001	< 0.001	< 0.001	< 0.001	< 0.001	< 0.001
**EPR**	Mean	0	0	0	0	0	0
	SD	1	−1	0	0	−1	−1
	Min	−1	0	0	0	0	1
	Max	6	−2	−2	−1	−2	−1
	Median	0	0	0	−1	0	0
	*P*	0.232	0.721	0.284	0.394	0.861	0.563
**ONL**	Mean	−10	−6	−4	−10	−5	−6
	SD	0	−5	−2	2	0	−3
	Min	−9	2	3	−3	−2	−3
	Max	−27	−116	−62	−33	−31	−68
	Median	−8	−5	−5	−10	−6	−7
	*P*	< 0.001	< 0.001	0.008	< 0.001	0.001	< 0.001
**OPL**	Mean	0	1	−1	3	−1	1
	SD	−1	1	1	4	0	1
	Min	10	12	−3	−1	−1	−3
	Max	−9	3	2	4	−8	2
	Median	1	1	−2	2	−2	0
	*P*	0.388	0.526	0.147	0.015	0.119	0.187
**INL**	Mean	1	−1	0	1	−1	0
	SD	2	1	1	2	0	0
	Min	0	5	−1	−2	−1	−3
	Max	10	5	6	13	−1	1
	Median	1	−1	−1	1	−1	1
	*P*	0.915	0.110	0.803	0.404	0.144	0.542
**IPL**	Mean	0	−2	−2	−2	−2	−2
	SD	0	0	1	0	0	0
	Min	2	6	−4	−3	0	−2
	Max	2	−1	−4	−1	1	−1
	Median	−2	−2	−1	−1	−3	−2
	*P*	0.156	< 0.001	< 0.001	< 0.001	< 0.001	< 0.001
**GCL**	Mean	−1	−4	−4	−3	−4	−4
	SD	0	1	1	−1	0	0
	Min	0	12	−6	−1	−1	−2
	Max	−3	−2	−2	−7	−8	−5
	Median	−1	−4	−3	−2	−2	−4
	*P*	0.010	< 0.001	< 0.001	< 0.001	< 0.001	< 0.001
**RNFL**	Mean	−1	−3	−3	−2	0	−2
	SD	0	−1	0	−1	1	0
	Min	0	3	−6	0	1	−3
	Max	2	−4	−7	−3	5	−4
	Median	0	−3	−2	−2	−1	−2
	*P*	0.020	< 0.001	< 0.001	< 0.001	< 0.001	< 0.001
**ALL**	Mean	−12	−13	−12	−13	−11	−12
	SD	1	−1	−1	−2	−2	−1
	Min	−34	−24	−17	−17	−13	−14
	Max	−22	−5	−29	−15	−11	−15
	Median	−8	−10	−12	−12	−13	−11
	*P*	< 0.001	< 0.001	< 0.001	< 0.001	< 0.001	< 0.001

mt: thickness of retinal layers at the foveal location (1 mm diameter). 0.3MT: mean thickness of retinal layers at the macular location (3 mm diameter). 0.3S: retinal layers thickness 3 mm superior to the fovea. 3I: thickness of retinal layers 3 mm inferior to the fovea. 3N: thickness of retinal layers 3 mm nasal to the fovea. 3T: thickness of retinal layers 3 mm temporal to the fovea. ORL, outer retinal layers; IRL, inner retinal layers; RPE, retinal pigment epithelium; ONL, outer nuclear layer; OPL, outer plexiform layer; INL, inner nuclear layer; IPL, inner plexiform layer; GCL, ganglion cell layer; RNFL, retinal nerve fiber layer; ALL, total retinal thickness.

The central foveal region (1 mm) exhibited a significant thickening in the IRL (*p* < 0.001), ONL (*p* < 0.001), GCL (*p* = 0.010), RNFL (*p* = 0.020), and ALL (*p* < 0.001). The other layers showed not statistically significant or negligible variations.

The superior parafoveal region (3 mm) presented a substantial increase in the IRL (*p* < 0.001), ONL (*p* < 0.001), IPL (*p* < 0.001), GCL (*p* < 0.001), RNFL (*p* < 0.001), and ALL (*p* < 0.001).

Additionally, a statistically significant thinning in the ORL (*p* = 0.002) was present in this region. The other layers showed not statistically significant or negligible variations.

The inferior parafoveal region (3 mm) displayed a statistically significant thickening in the IRL (*p* < 0.001), ONL (*p* = 0.008), IPL (*p* < 0.001), GCL (*p* < 0.001), RNFL (*p* < 0.001), and ALL (*p* < 0.001). A statistically significant reduction in the ORL thickness (*p* < 0.001) was also demonstrated. The other layers showed not statistically significant or negligible variations.

The nasal parafoveal region (3 mm) showed a statistically significant increase in the IRL (*p* < 0.001), ONL (*p* < 0.001), IPL (*p* < 0.001), GCL (*p* < 0.001), RNFL (*p* < 0.001), and ALL (*p* < 0.001). A meaningful decrease in the ORL (*p* = 0.002) and OPL (*p* = 0.015) was also shown.

The other layers showed not statistically significant or negligible variations.

The temporal parafoveal region (3 mm) displayed a statistically significant increase in the IRL (*p* < 0.001), ONL (*p* = 0.001), IPL (*p* < 0.001), GCL (*p* < 0.001), RNFL (*p* < 0.001), and ALL (*p* < 0.001). A statistically significant decrease in the ORL (*p* < 0.001) was also demonstrated.

The other layers showed not statistically significant or negligible variations.

The average of the five considered regions (foveal and parafoveal) demonstrated a statistically significant thickening of the IRL (*p* < 0.001), ONL (*p* < 0.001), IPL (*p* < 0.001), GCL (*p* < 0.001), RNFL (*p* < 0.001), and ALL (*p* < 0.001). A statistically significant thinning in the ORL layer (*p* < 0.001) was also displayed. The other layers showed not statistically significant or null variations.

In addition, PR layer showed a statistically significant reduction in the 4 parafoveal regions (*p* < 0.001), however not in the central foveal region (Tables 3, 4).

**TABLE 3:** Thickness of photoreceptors layer (μm).

		PRE						POST					
		mt	3S	3I	3N	3T	3MT	mt	3S	3I	3N	3T	3MT
**PR**	Mean	70	66	66	67	67	66	69	65	64	65	65	65
	SD	4.2	2.4	3.1	3.1	2.8	2.6	3.9	2.5	2.2	2.6	2.7	2.3
	Min	61	62	62	62	61	62	61	61	61	61	56	61
	Max	79	73	84	82	76	77	82	71	71	75	73	73
	Median	69	66	66	66	66	66	69	65	64	65	65	65

mt: thickness of retinal layers at the foveal location (1 mm diameter). 3MT: mean thickness of retinal layers at the macular location (3 mm diameter). 0.3S: retinal layers thickness 3 mm superior to the fovea. 3I: thickness of retinal layers 3 mm inferior to the fovea. 3N: thickness of retinal layers 3 mm nasal to the fovea. 3T: thickness of retinal layers 3 mm temporal to the fovea.

**TABLE 4:** Difference in thickness (μm) of photoreceptors layer pre/post-operation.

		Δ PRE/POST					
		Mt	3S	3I	3N	3T	3MT
**PR**	Mean	1	1	2	2	2	2
	SD	0.3	0.1	0.9	0.5	0.1	0.3
	Min	0	1	1	1	5	1
	Max	−3	2	13	7	3	5
	Median	−1	1	2	1	1	1
	*P*	0.236	< 0.001	< 0.001	< 0.001	< 0.001	< 0.001

mt: thickness of retinal layers at the foveal location (1 mm diameter). 0.3MT: mean thickness of retinal layers at the macular location (3 mm diameter). 0.3S: retinal layers thickness 3 mm superior to the fovea. 3I: thickness of retinal layers 3 mm inferior to the fovea. 3N: thickness of retinal layers 3 mm nasal to the fovea. 3T: thickness of retinal layers 3 mm temporal to the fovea.

## Discussion

In recent years phacoemulsification for cataract treatment has been gradually improved, becoming a minimally invasive procedure that involves only the anterior segment of the eye.

However, post-operative retinal alterations can have serious consequences, such as cystoid macular edema, or they may present sub-clinically and seem to have no effect on visual outcome.

The precise cause and mechanism behind the fundus changes following emulsification of cataractous lenticular nucleus remain unclear. Several factors have been suggested, including vascular instability, vitreomacular traction, ocular hypotony, and increased light exposure (26).

Some studies indicate that post-operative inflammation may significantly contribute to the evelopment of retinal alterations (27–30).

Numerous factors related to phacoemulsification can affect ocular structures. Ultrasonic energy and fluid dynamics generate mechanical effects, albeit slight, that result in inflammation, compression, and oxygen drop in the surrounding tissues. Each step of this procedure can induce direct tissue changes and immediate pressure variations. Additionally, turbulent fluid flow exerts radiating pressure impacts similar to a shockwave and a small jet, directly affecting the anterior chamber structures and spreading in all directions (9).

Even if asymptomatic, these micro-alterations can be detected with optical coherence tomography (OCT).

Although several studies have been conducted in recent years to verify changes in macular thickness after cataract surgery, their conclusions were not in agreement (14–19, 25, 31, 32).

The present study focuses on changes one month after phacoemulsification, a significant increase in thickness is observed in many retinal layers (IRL, ONL, IPL, GCL, RNFL) both at the central foveal and parafoveal regions, leading to a statistically significant increase in ALL. This finding is consistent with the most recent studies that have examined retinal modifications in terms of thickness following cataract surgery (14–19, 25, 31, 32). However, among these studies, only one segmented the retina and examined the modifications individually.

Nevertheless, this research was limited by the modest number of examined eyes and segmenting the retina into the following layers: RPE, ONL, OPL, INL, IPL, GCL, RNFL (25).

This segmentation excluded the retinal structures between the RPE and ONL, which include photoreceptors and their connections with inner layers (33). Our study introduced further segmentation of the retina into IRL and ORL. The latter group includes all structures between RPE and the ELM, including PRL. Then we obtained the PRL from the ORL, and the results revealed that the ORL, in particular the PRL, is the only segment to show a statistically significant thinning in the 4 parafoveal regions, with no changes in the central foveal region.

This result, observed for the first time in this study, is in contrast with the findings of other studies and may indicate inflammation-induced suffering of the PRL, following phacoemulsification. This structural alteration was not evident during clinical examination, and the sparing of the central foveal region could explain the absence of clinical influence.

It is known that post-operative inflammatory effects can cause free radicals, growth factors and prostaglandins release, which could be leading factors for post-operative retinal alterations (5, 24, 34). By the literature, surgical wounds trigger releasing of prostaglandins into the aqueous humor and blood-aqueous barrier damage, with consequential start of an inflammatory cascade and production of other inflammatory mediators in the aqueous humor and their dispersion into the vitreous cavity. Posterior segment inflammation, consecutive to the anterior segment ones, disrupts both inner and outer blood-retinal barrier (35), inducing choroidal thickness and Vascularity Index increase at the first postoperative month (35–37). Nevertheless, lacking strong evidence, the exact mechanism of retinal structure changes and their impact on the ocular fundus–whether beneficial or harmful–remains unclear.

Menapace et al. (38) published a study involving 120 eyes from 60 patients, with complete follow-up data for 56 patients. The participants were divided into two groups: those undergoing femtosecond laser-assisted cataract surgery and those undergoing manual cataract surgery. The findings indicated similar patterns of macular thickness and volume increase, with no statistically significant differences between the two groups. Our results are consistent with those of the authors, despite our focus on individual retinal layers, to achieve a meticulous assessment of retinal changes (38).

Schwarzenbacher et al. (39) conducted a study involving 112 eyes from 56 patients, also divided into femtosecond laser-assisted and manual cataract surgery groups. They evaluated changes over postoperative periods of 1 week, 3 weeks, 6 weeks, and 18 months, focusing on the inner nuclear layer (INL), outer plexiform layer (OPL), outer nuclear layer (ONL), photoreceptor (PR) layer, and total retinal thickness. Notably, a significant decrease in PR thickness was observed 1 week post-surgery across all zones. Conversely, the other evaluated layers and the overall retina exhibited an increase in thickness during the initial weeks, remaining significantly elevated 18 months post-surgery in all zones. Our findings align with those of the authors in the early postoperative period; however, our study utilized a larger sample size of 70 eyes from a total of 70 subjects and assessed all individual retinal layers (39).

Großpötzl et al. (40) investigated a cohort of 41 patients who underwent uneventful cataract surgery, with evaluations conducted preoperatively and at 1 day, 1 month, and 3 months postoperatively. The authors focused on all layers of the inner retina, including the OPL, ONL, and total retinal thickness. They reported a decrease in retinal thickness on the first postoperative day, followed by a significant increase at 1 month and a subsequent reduction at 3 months. Our results are consistent with these findings at 1 month post-surgery; however, the strength of our research lies in its comprehensive assessment of all retinal layers. Unlike the authors, we observed a statistically significant decrease in the OPL and PR layer at 1 month post-surgery (40).

Recently, Balog et al. (41) published a study involving 102 eyes from 79 consecutive subjects without any other ocular or systemic diseases who underwent cataract surgery. The authors reported a statistically significant increase in nearly all retinal layers and a decrease in OPL at 7, 30 and 90 days post-phacoemulsification. This finding aligns with our results; we also observed thickening in nearly all retinal layers and thinning of the OPL. The authors suggested that the decrease might be attributed to PR loss. While we cannot confirm this hypothesis, our study specifically focused on the PR layer and found a statistically significant reduction. This decrease may indeed be explained by PR loss; however, our results indicate a reduction in thickness that could also be attributed to structural cellular changes within the PR layer (41).

In conclusion, our study, conducted on 70 eyes of 70 patients scheduled for cataract surgery supports the existing literature, highlighting an increase in retinal thickness following the procedure, and also reveals a previously unobserved reduction in the thickness of the ORL and PRL, observed through a more detailed segmentation of retinal layers than ever before. Due to the retrospective nature of this study, longer follow up visit were not available, because nor morphological signs of retinal changes by OCT neither best corrected visual acuity reduction were present. For the same reason, only the routine pharmacological treatment with NSAID drops was applied to these patients.

The lack of longer follow-up to strengthen our observation represents a limitation of this study. Nevertheless, our results provide a basis for further studies that may provide a pathophysiological explanation for this phenomenon, contributing to an understanding of post-phacoemulsification complications affecting the retina.

## Data Availability

The raw data supporting the conclusions of this article will be made available by the authors, without undue reservation.
